# Targetable alterations and personalized treatment in ameloblastoma: results from a prospective observational precision oncology study

**DOI:** 10.1038/s41698-026-01527-6

**Published:** 2026-06-12

**Authors:** Elena Hofmann, Shady Abu-Sirhan, Fabian Elsholtz, Iris Piwonski, Kilian Kreutzer, Christian Doll, Maria Joosten, Markus Möbs, Chia-Jung Busch, Benjamin Fenske, Maren Knödler, Ana Pestana, Ariel Hirschhorn, Max Heiland, Ulrich Keller, Ulrich Keilholz, Konrad Klinghammer, Damian T. Rieke, Max Schmidt

**Affiliations:** 1 Department of Oral and Maxillofacial Surgery, Charité – Universitätsmedizin Berlin, Corporate Member of Freie Universität Berlin and Humboldt-Universität zu Berlin, Berlin, Germany; 2 German Cancer Consortium (DKTK), Partner site Berlin a partnership between DKFZ and Charité – Universitätsmedizin Berlin, Berlin, Germany; 3 National Center for Tumor Diseases (NCT), NCT Berlin, a partnership between DKFZ, Charité – Universitätsmedizin Berlin, Berlin Institute of Health at Charité (BIH) and Max Delbrück Center, Berlin, Germany; 4 Department of Radiology, Charité – Universitätsmedizin Berlin, Corporate Member of Freie Universität Berlin and Humboldt-Universität zu Berlin, Campus Benjamin Franklin, Berlin, Germany; 5 Institute of Pathology, Charité – Universitätsmedizin Berlin, Corporate Member of Freie Universität Berlin and Humboldt-Universität zu Berlin, Berlin, Germany; 6 Department of Otorhinolaryngology, University Medicine Greifswald, Greifswald, Germany; 7 Charité Comprehensive Cancer Center, Charité – Universitätsmedizin Berlin, Corporate Member of Freie Universität Berlin and Humboldt-Universität zu Berlin, Berlin, Germany; 8 Department of Cranio-Maxillofacial Surgery, Sheba Medical Center, Ramat Gan, Israel; 9 Department of Hematology, Oncology and Cancer Immunology, Charité – Universitätsmedizin Berlin, Corporate Member of Freie Universität Berlin and Humboldt-Universität zu Berlin, Campus Benjamin Franklin, Berlin, Germany; 10 Max Delbrück Center for Molecular Medicine (MDC) in the Helmholtz Association, Berlin, Germany; 11 Cluster of Excellence ImmunoPreCept, Charité – Universitätsmedizin Berlin, Berlin, Germany

**Keywords:** Cancer, Oncology

## Abstract

Ameloblastomas are locally aggressive jaw tumors, primarily treated with radical surgery, which often results in significant functional and aesthetic morbidity. Innovative, personalized treatment strategies to reduce patient burden without compromising oncologic control remain lacking. This prospective observational study included comprehensive molecular profiling of 14 patients with ameloblastoma. Pathogenic mutations were identified in all cases, including alterations in *BRAF, SMO, HRAS, FGFR2*, and *PIK3CA*. Personalized treatment recommendations were made for 13 patients, and 11 received matched therapies: dabrafenib ± trametinib (*n* = 9), futibatinib (*n* = 1), or binimetinib (*n* = 1). Radiological tumor regression occurred in 10 of 11 treated patients. Four patients underwent surgery following neoadjuvant therapy, allowing for reduced resection extent and resulting in partial or complete pathological responses. These findings demonstrate frequent actionable alterations in ameloblastoma and clinically meaningful responses of targeted therapies. Incorporating precision oncology into standard care may facilitate personalized, less morbid surgery and improved outcomes in these rare tumors.

## Introduction

Ameloblastoma is a rare, locally aggressive odontogenic tumor that primarily affects the craniofacial skeleton, predominantly the mandible (80–90%)^1–3^. With a global incidence of approximately 1 per million per year, ameloblastomas are diagnosed in both pediatric and adult patients, with a low mean age at diagnosis of around 34 years^2^. Ameloblastomas are categorized into three histopathological subtypes: conventional or multicystic (75–86%), the most aggressive and recurrent; unicystic (5–22%); and peripheral (1.5%)^3–5^. Despite being classified as histopathologically benign, it exhibits locally aggressive and infiltrative growth, reminiscent of malignant neoplasms, often resulting in severe facial deformities and, in rare cases, malignant transformation.

Clinical diagnosis is frequently delayed due to the absence of symptoms in early stages, with many cases only identified once the tumor has reached an advanced size, causing severe jaw deformity, facial asymmetry, and functional impairment. Managing advanced ameloblastoma presents significant challenges for head and neck centers, as the current standard of care involves extensive surgery, typically requiring segmental mandibulectomy and microvascular bone flap reconstruction to achieve functional and aesthetic rehabilitation. Postoperative morbidities may involve facial asymmetry with impaired aesthetics, extensive scarring, lower lip numbness, tooth loss in the affected area, and reduced masticatory function^6^, affecting the quality of life in these often young patients.

Conservative surgical approaches like enucleation or marsupialization prioritize the preservation of the uninvolved jawbone, but carry higher risk of recurrence. For multicystic ameloblastoma, recurrence rates are as high as 41% following conservative surgery. In contrast, radical ablative resection leads to lower recurrence rates of ~8%, and is thus the preferred surgical approach for this histological subtype^7,8^. A meta-analysis reported a threefold higher recurrence risk with conservative versus radical surgery^9^. Radiotherapy and chemotherapy have no established role in the primary management and are reserved only for non-resectable, palliative^10^, or rare metastatic cases^11,12^. Thus, surgical resection remains the mainstay of treatment; however, there is an urgent need for innovative strategies to reduce morbidity without compromising oncologic outcome.

Comprehensive genomic profiling has characterized ameloblastomas as oncogene-driven, harboring mutations in *BRAF*, *SMO*, *FGFR2*, *KRAS*, *NRAS*, and *HRAS*, pointing to canonical and non-canonical *GLI* signaling as downstream effector^13–16^. *BRAF* and *SMO* mutations are reported as mutually exclusive, with *BRAF* V600E predominating in mandibular and *SMO* mutations enriched in maxillary ameloblastomas^15,17^. Present in over 50% of ameloblastoma, *BRAF* V600E is a key oncogenic driver^18^, with BRAF ± MEK-inhibition demonstrating high response rates (~70%) and rapid tumor regression in other cancers^18–21^. Emerging case reports suggest similar potential in *BRAF* V600E-mutated ameloblastoma, where neoadjuvant therapy may reduce tumor size, surgical extent, and postoperative morbidity^22,23^.

The advent of targeted therapies holds promise for advancing ameloblastoma management and complementing the only available surgical treatment options. However, standardized precision oncology workflows for ameloblastomas are lacking, and the role of neoadjuvant targeted therapies warrants further investigation.

This study assesses targetable alterations in ameloblastoma and the effect of molecularly guided therapies, both within and beyond classical *BRAF* V600E mutations, applying a precision oncology workflow in a prospective observational setting. The primary objective was to assess radiological and pathological responses to personalized therapy, particularly in the pre-surgical setting. In this context, tumor size reduction may facilitate less extensive surgery with lower morbidity, without compromising long-term disease control.

## Results

### Patient characteristics

**Fig. 1:**
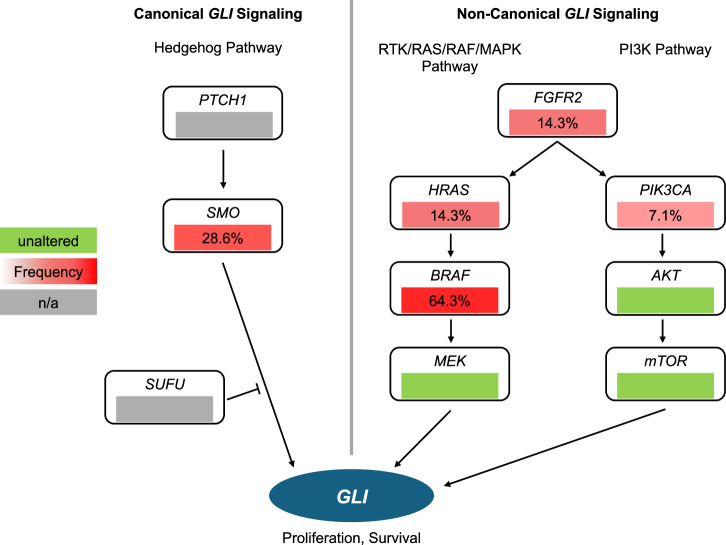
Schematic of canonical and non-canonical *GLI* signaling across the Hedgehog, RTK/RAS/RAF/MAPK, and PI3K pathways. Color coding reflects mutation status and frequency among 14 patients regardless of pathogenicity (green: wild type; red: mutation detected, with shade indicating frequency). Pathway genes not covered by the assay are shown in grey (n/a).

**Fig. 2:**
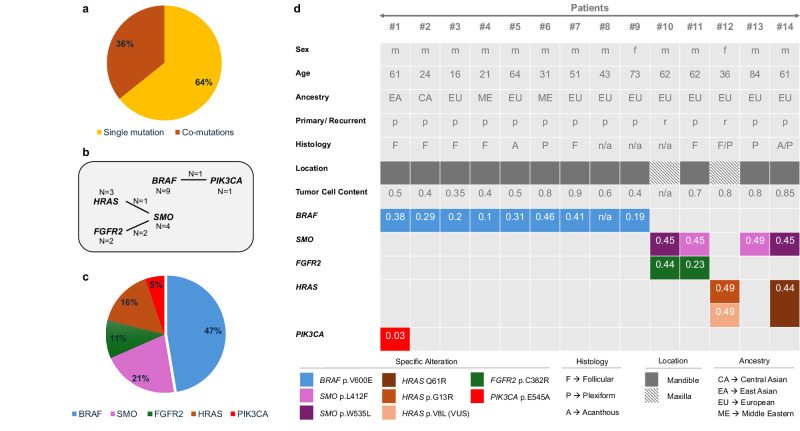
Clinicogenomic details and mutational complexity in the study cohort. Figure showing the **a** proportion of patients with single mutation or co-mutation, **b** the mutational complexity across all patients, **c** the mutation frequency relative to the total number of detected mutations in the cohort. **d** Case-specific representation of sex (*m* = male [85.7%]; *f* = female [14.3%]), age (in years [median 56 years; IQR 32–62]), patient-reported ancestry (EU = European [71.4%]), and disease status (*p* = primary [85.7%]; *r* = recurrent [14.3%]) at the time of genomic profiling, tumor cell content, mutation status with variant allele frequency (VAF), histology, and anatomical location. All cases exhibited the multicystic subtype. Each column represents an individual case, with color coding indicating the respective mutation, histological classification, and primary tumor site.

The cohort included 14 patients with ameloblastoma: 12 males (85.7%) and 2 females (14.3%), aged between 16 and 84 years (median age: 56 years; IQR: 32‒62 years) at the time of genomic profiling (Fig. 1, Fig. 2a–d). All tumors were multicystic ameloblastoma. Histological subtypes included follicular (*n* = 6), plexiform (*n* = 2), acanthomatous (*n* = 1), follicular/plexiform (*n* = 1), and acanthomatous/follicular (*n* = 1); three cases were uncategorized. Self-reported ancestry was predominantly European (71.4%), with remaining patients of Middle Eastern, Central Asian, or East Asian. Patients presented with symptoms such as jaw swelling, pain, or difficulty eating. Advanced tumor size led to facial disfigurement with significant aesthetic and function impairments.

Twelve patients presented with newly diagnosed, treatment-naive mandibular ameloblastomas. Of these, eight were located in the left mandible, extending between the mandibular body and ramus, and three involved the right molar region. Patient #2 presented with a large tumor, spanning from the left to the right angle of the jaw, involving all teeth from the left second molar to the right third molar.

Two patients presented with refractory/recurrent maxillary ameloblastomas. Patient #10 with recurrent ameloblastoma of the right maxilla underwent multiple resections (2004, 2013) and radiotherapy (60 Gy, 2016). In 2023, the patient presented with a recurrence extending into the right nasal cavity, nasopharynx, superior ethmoid cells, sphenoid sinuses, impairing nasal breathing. Patient #12, diagnosed with ameloblastoma of the left maxilla in 2007, had seven resections over the next 17 years. In 2024, recurrence in the left temporal fossa showed dural contact and retroorbital extension, causing mild displacement of the left eyeball.

### Genomic alterations and treatment strategies

Pathogenic mutations in oncogenes were identified in all patients (Fig. 2a–d). Single mutations were detected in 9 tumors, while 5 harbored 2 mutations (Fig. 2a). The *BRAF* V600E was most common (9/14; 64%), followed by *SMO* (*n* = 4), *FGFR2* (*n* = 2), *HRAS* (*n* = 2), and one case with a *PIK3CA* co-mutation. In total, 19 mutations spanning 8 distinct variants were detected. Oncogenic variants included *BRAF* V600E, *SMO* W535L*/* L412F, *FGFR2* C382R, and *PIK3CA* E545A. *HRAS* variants included G13R and Q61R (likely pathogenic) and V8L (VUS). Variant allele frequencies (VAF) ranged from 2.7% to 49% and corresponded to reported tumor cell content. In the case of *PIK3CA*, a lower VAF indicated subclonality. All detected mutations mapped to components of canonical or non-canonical *GLI* signaling cascade, highlighting a potentially convergent pathway dependency in ameloblastoma (Fig. 1). *BRAF* and *SMO* mutations were mutually exclusive (Fisher’s exact test, *p* = 0.005), with three of four *SMO*-mutated tumors harboring co-mutations in *FGFR2* or *HRAS*. No significant association with ancestry or tumor location was observed for any gene.

**Fig. 3:**
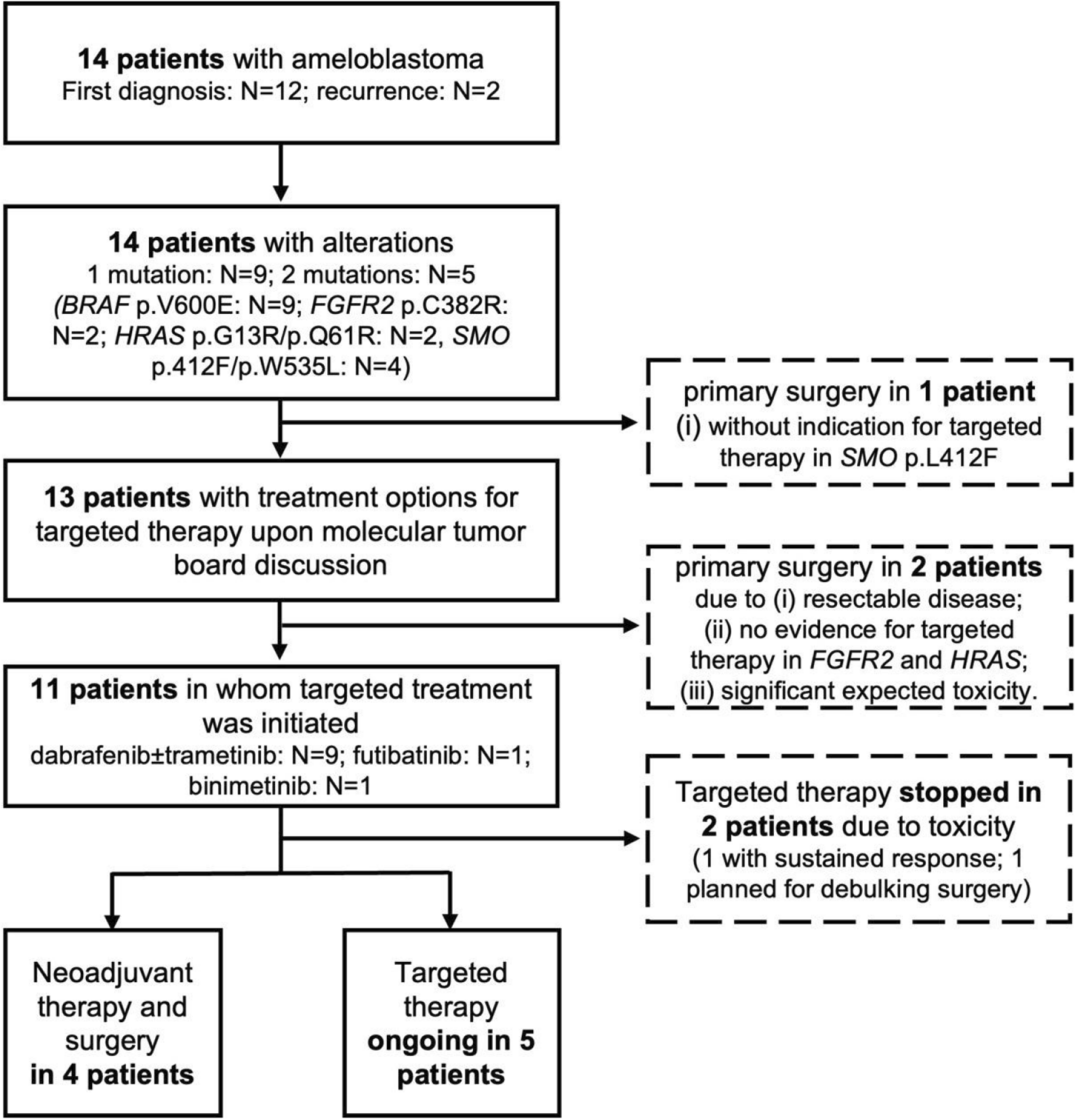
Flow chart detailing the patient cohort.

Three patients underwent primary surgical treatment, and 11 received targeted therapy (Fig. 3). Among 9 patients with *BRAF* V600E mutations, 8 received dabrafenib (150 mg twice daily) plus trametinib (2 mg daily); 1 patient opted for dabrafenib monotherapy (150 mg twice daily) due to cost constraints, despite recommendation of combined BRAF/ MEK inhibition. One patient with an *FGFR2* mutation received futibatinib (20 mg once daily), and one with an *HRAS* mutation was treated with binimetinib (45 mg twice daily). At time of analysis, four patients had undergone surgical resection following targeted therapy.

### Targeted therapy activity

**Fig. 4:**
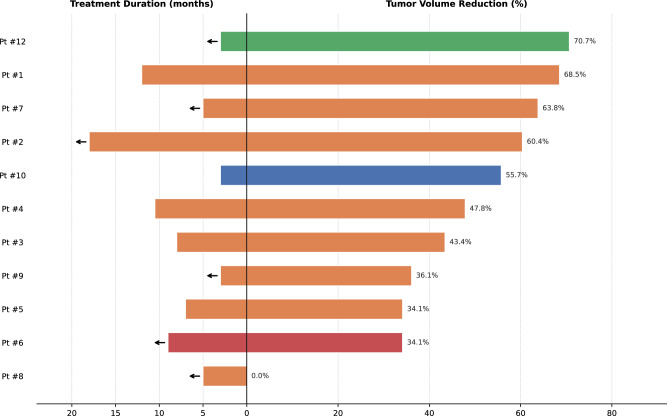
Treatment duration and tumor volume reduction after personalized targeted therapy. Treatment duration in months (left) and tumor volume reduction in percentage (right) based on MRI volumetry in 11 ameloblastoma patients treated with molecularly matched targeted therapies. Numbers (#) denote individual patients. Colors indicate the administered treatment: green = binimetinib; blue = futibatinib; orange = dabrafenib + trametinib; red = dabrafenib. Tumor volume reduction was calculated by comparing MRI scans obtained prior to the initiation of targeted therapy with the MRI performed after targeted therapy completion. For patients in whom targeted treatment was ongoing at the data cut-off (indicated by arrow), the most recent follow-up MRI was used.

Radiological tumor volume reduction of 34% to 71% was observed in 10 of 11 patients receiving targeted therapies for 3 to 18 months (Fig. 4). No new or unexpected adverse events occurred. Dabrafenib + trametinib was well tolerated, with only transient grade II toxicities (e.g., peripheral edema, elevated creatine kinase). Futibatinib caused hyperphosphatemia, gastrointestinal disturbances, and hepatotoxicity, necessitating dose reduction and eventual discontinuation. Binimetinib-related toxicities included rash, renal impairment, and elevated creatine kinase, prompting early therapy cessation.

Among nine patients with BRAF V600E-mutated ameloblastoma, eight exhibited radiological tumor regression and reduced contrast enhancement under dabrafenib ± trametinib (Supplementary Fig. S1). Patient #8 showed no response with unchanged signal intensity and tumor size after three months.

Patient #10, with a refractory *FGFR2*-mutated tumor, received futibatinib for three months, achieving a 56% tumor volume reduction and sustained signal intensity decrease (Supplementary Fig. S2a–c) over six months, along with improved nasal airflow. In patient #12, with *HRAS* mutation and multiple recurrences, three months of binimetinib resulted in a 71% tumor volume reduction in the orbit and infratemporal fossa and reduced signal intensity, enabling planning for a tissue-preserving CADCAM-guided debulking surgery (Supplementary Fig. S3a–d).

### Response to neoadjuvant BRAF-targeted therapy and surgical treatment

**Fig. 5:**
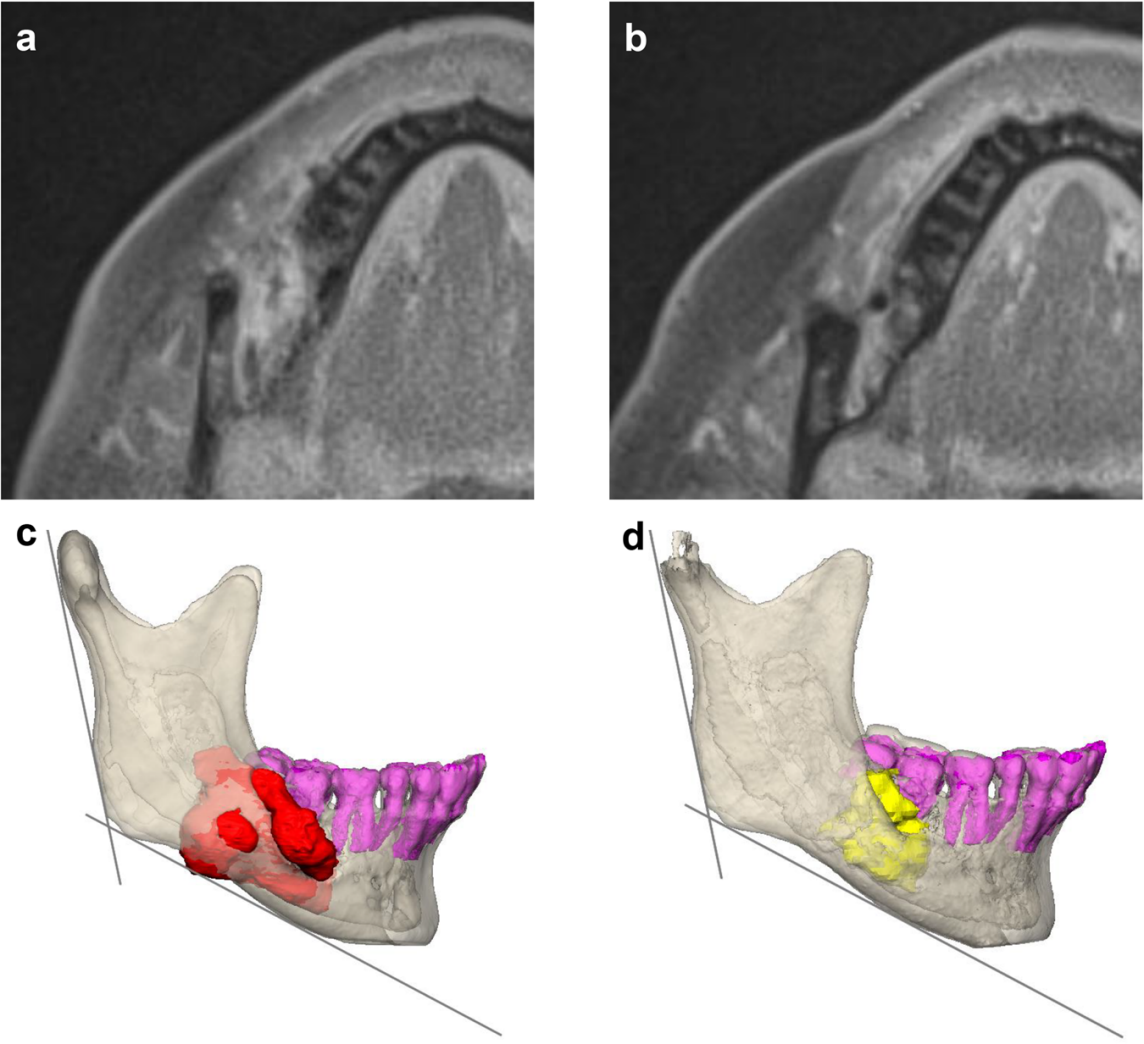
Case presentation demonstrating changes in ameloblastoma extent following neoadjuvant targeted therapy. Patient #1 presented with *BRAF* V600E-mutated ameloblastoma of the right mandible and was started on neoadjuvant targeted therapy. The reduction in tumor size, reduced signal intensity and signs of re-ossification are illustrated by **a**, **b** MRI (T1-weighted sequence with fat saturation) following intravenous contrast administration and **c**, **d** virtual, three-dimensional segmentations, **a**, **c** prior to and **b**, **d** following neoadjuvant targeted therapy. red= tumor before neoadjuvant therapy; yellow = tumor after neoadjuvant therapy; purple = teeth).

**Fig. 6:**
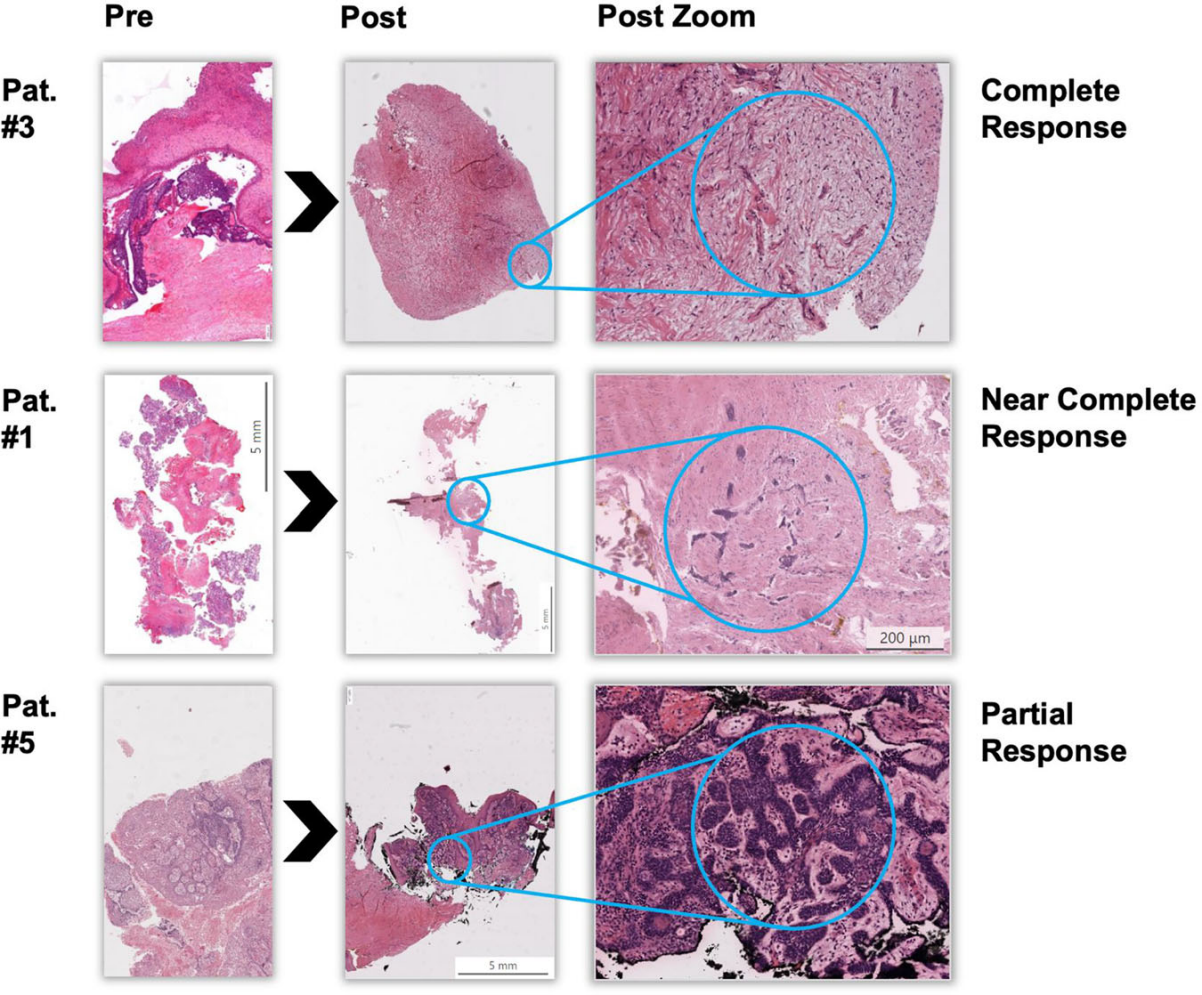
Pathological assessment of tumor regression phenotypes following neoadjuvant dabrafenib plus trametinib treatment. Three distinct histopathological regression patterns observed in this cohort are illustrated: complete, near complete, and partial response. Representative hematoxylin and eosin (H&E) slides from pre- (Pre) and post-treatment (Post) specimens are shown. Microscopic zooms of selected post-treatment regions are graphically indicated in blue.

As of March 2025, four patients (#1, #3, #4, and #5) underwent surgical resection following 7 to 12 months (median: 9.25 months) of *BRAF*-directed therapy with dabrafenib + trametinib. MRI showed a mean tumor volume reduction of 48.5% (95% CI: 34.1–68.5%), and reduced signal intensity prior to surgery, with radiological re-ossification evident in three patients (#1, #4, #5) (Fig. 5a–d). The sole *PIK3CA*-comutated tumor (#1) showed the highest volumetric response (68.5% over 12 months) compared to *PIK3CA* wild-type tumors (median 43.4% over 8 months), though formal testing is precluded by cohort size. Histopathological assessment of the resection specimens revealed one complete, two near complete, and one partial remission (Fig. 6).

Neoadjuvant therapy reduced the resection complexity score to ≤5 with a median reduction of 7 points (Table 1), and all patients underwent enucleation with an intraoral approach following preserving principles. In patients #1 (Fig. 5a–d) and #5, two teeth adjacent to the tumor were extracted. In patients #3 and #4, the retained wisdom tooth was removed, and osteosynthesis was performed for stability. Inferior alveolar nerve neurolysis was feasible in patients #4 and #5. Patient #5 required soft tissue resection due to a soft tissue fistula. Postoperative hypoesthesia of the ipsilateral inferior alveolar nerve was observed in all patients. MRI-based follow-up at 12 months postoperatively was available for all patients (*n* = 4) with no recurrence detected.

**Table 1:** Changes in the resection complexity score after neoadjuvant BRAF-targeted therapy

	Pre-neo RCS vs. post-neo RCS											
	Patient #1			Patient #3			Patient #4			Patient #5		
Category (Score min-max)	Pre-neo RCS	Post-neo RCS	Δ	Pre-neo RCS	Post-neo RCS	Δ	Pre-neo RCS	Post-neo RCS	Δ	Pre-neo RCS	Post-neo RCS	Δ
**Bone resection (0–2)** Segmental resection (0) Non-segmental resection (1) No bone resection (2)	2	0	2	2	0	2	2	0	2	2	0	2
**Soft tissue involvement (0–2)** Extensive soft tissue resection (0) Fistula resection (1) No soft tissue resection (2)	1	0	1	1	0	1	1	0	1	2	1	1
**Involvement of inferior alveolar nerve (0–2)** Nerve resection (0) Neurolysis (1) No intervention on nerves (2)	2	0	2	2	0	2	2	1	1	2	1	1
**Involvement of teeth (0–2)** Extraction of ≥ 2 teeth (0) Root tip resection or extraction of 1 tooth (1) No intervention on teeth (2)	2	2	0	2	1	1	2	1	1	2	2	0
**Surgical approach (0–2)** Extra- and intraoral (0) Extraoral (1) Intraoral (2)	2	0	2	2	0	2	2	0	2	2	0	2
**Combined RCS (0–10)**	**9**	**2**	**7**	**9**	**1**	**8**	**9**	**2**	**7**	**10**	**4**	**6**

This table details the resection complexity score in patients who completed neoadjuvant BRAF-targeted therapy and underwent surgical therapy (*n* = 4). The reduction in resection complexity score was compared based on the virtual surgical plan prior to neoadjuvant therapy and after completion of targeted therapy. The combined RCS and Pre/Post Delta are depicted in bold as the sums of the sub-category scores.

*RCS* resection complexity score, *pre-neo* pre-neoadjuvant, *post-neo* post-neoadjuvant.

### Outcome after primary radical resection

Three patients (#11, #13, and #14) underwent tailored primary surgical therapy without prior matched systemic therapy. Patient #11 with a follicular ameloblastoma of the left mandible (regio 37-38) underwent enucleation with one tooth extracted under outpatient general anesthesia. Postoperatively, hypoesthesia of the left inferior mandibular nerve was observed. Patient #13, with a 68 mm plexiform ameloblastoma, underwent segmental mandibulectomy with free-flap fibula reconstruction and CADCAM patient-specific implant. Postoperative outcomes included hypoesthesia and an extraoral scar. The patient is scheduled for implant-supported dental rehabilitation. Patient #14 underwent a continuity-preserving jaw resection using patient-specific cutting guides and inferior alveolar nerve reconstruction using a sural nerve autograft. An intraoperative mandibular fracture required reposition and fixation using osteosynthesis material through an extraoral approach. Postoperative outcomes included inferior alveolar nerve anesthesia and an extraoral scar.

Of these three patients receiving primary surgery, one patient (#11) exhibited a local recurrence within the observation period and was being evaluated for *FGFR*-targeted therapy. The other two patients remained disease-free at 10 and 17 months postoperatively.

## Discussion

This study demonstrates the clinical value of comprehensive genomic profiling in ameloblastoma, revealing a mutation landscape dominated by targetable alterations, including *BRAF* V600E (64% of cases), *FGFR2*, and *HRAS* (14.3% each). Our findings align with prior reports of recurrent alterations in canonical and non-canonical *GLI* signaling^13–16,24^, reinforcing ameloblastomas oncogene-driven yet molecularly simple profile. The high prevalence of *BRAF* V600E supports the integration of molecularly guided therapies into clinical practice, offering the potential to reduce surgical morbidity without increasing the risk of recurrence.

We present the application of a precision oncology workflow aligned with current guidelines of the National Center for Tumor Diseases (NCT) and Centers for Personalized Medicine (CPM)^25–28^, incorporating tumor genomic profiling, evidence-based annotation of actionable alterations, and personalized treatment recommendations from a multidisciplinary molecular tumor board. Treatment recommendations were based on predictive biomarkers associated with response to approved targeted therapies and patient-specific factors. Grynberg et al. reported radiological responses in 14 *BRAF* V600E-mutant ameloblastoma patients treated with dabrafenib ± trametinib (NCT/CPM evidence level M1B)^23^. Based on these outcomes and favorable efficacy and toxicity profile, dual BRAF/MEK inhibition was recommended upfront for *BRAF* V600E-mutated cases to reduce tumor size and enable de-escalated, less morbid surgery.

Building on this, other targetable alterations merit consideration. FGFR2-directed therapies are approved for *FGFR*-fusion-positive malignancies^29^, but may also be effective in *FGFR2*-mutant tumors^30^. Two previously reported cases of refractory ameloblastoma treated with FGFR2-targeted therapy—lenvatinib (*FGFR2* p.Y375C)^31^ and erdafitinib (*FGFR2* p.V395D)^32^– demonstrated partial radiologic responses (NCT/CPM evidence level M1C), but were associated with significant toxicities. In this cohort, *FGFR2* mutations (p.C382R) were identified in patients #10 and #11. In patient #10, where R0 resection was unfeasible, *FGFR*2-directed therapy with futibatinib was initiated. Patient #11 underwent primary surgery.

Mutations in *RAS* genes currently lack established targeted therapy options. MEK inhibitors like binimetinib have shown benefit in Ras-driven malignancies, including Langerhans cell histiocytosis (NCT/CPM evidence level M2B)^33^, and *NRAS*-mutated tumors in the NCI-MATCH trial, comprising one case of malignant ameloblastoma with a partial response (NCT/CPM evidence level M1C)^34^. These findings suggest that MEK inhibition may be viable in carefully selected *RAS*-mutated cases. Binimetinib was recommended for patient #12 with unresectable ameloblastoma. Primary resection was recommended for patient #14.

Other, less frequent alterations present distinct challenges. Oncogenic *PIK3CA* mutations predict response to alpelisib in hormone receptor-positive breast cancer, but evidence in other tumors is limited^35^. PI3K inhibitors are associated with notable toxicity risks (e.g., hyperglycemia, hypertension, electrolyte imbalances, acute kidney injury)^36,37^ and may be less effective in tumors with *RAS/RAF/MEK* pathway co-mutations linked to resistance^38–40^. In patient #1, the low VAF of 2.4% in the *PIK3CA* mutation suggested subclonality, and targeted therapy was not recommended due to limited evidence and toxicity risk.

Pathogenic *SMO* mutations in the Hedgehog pathway, characteristic of ameloblastoma and reflecting canonical GLI signaling^13,15,17,41^, likely confer resistance to approved SMO-inhibitors (vismodegib, sonidegib)^42,43^. Though arsenic-trioxide (ATO) may suppress GLI1-mediated downstream Hedgehog signaling in vitro^15,44^, its clinical activity remains uncertain, and *SMO* mutations did not inform treatment recommendations in this cohort. Furthermore, co-occurrence of *SMO* with *FGFR2* or *HRAS* mutations warrants investigation of concurrent Hedgehog signaling as a potential resistance mechanism to matching targeted therapies, and vice versa. Further research is needed to clarify the therapeutic implications of these alterations in ameloblastoma. However, the here described activity of targeted treatment even in the presence of co-occurring alterations does not support clinically relevant resistance mechanisms.

Neoadjuvant therapy with dabrafenib ± trametinib in *BRAF* V600E-mutated ameloblastoma resulted in notable radiological and pathological responses within 7 to 12 months, with tumor volume reductions of 34.1% to 68.5%, enabling enucleation as a function-preserving surgery option. However, individual treatment duration should be considered for larger tumors or gradual responses. Individual therapy duration may be safely extended if ongoing tumor regression can be observed via serial imaging. This prolonged treatment window is particularly relevant given the slow to moderate growth kinetics of ameloblastoma, with reported mean Ki-67 indices of ~40% and mean annual growth rates of 40‒88%^45–47^. The high tumor volume reduction observed in one BRAF/ PIK3CA-co-mutated tumor was unexpected, given that PI3K pathway activation represents an established bypass resistance mechanism to BRAF/MEK inhibition in melanoma; the reasons for this discordance requires investigation in larger ameloblastoma cohorts^40,48^.

While evidence for BRAF-directed therapy is growing^22,23^, our study also highlights the potential and challenges of non-BRAF-targeted treatments. Patients with *FGFR2* and *HRAS* mutations showed radiological responses with futibatinib and binimetinib, but faced toxicity-related discontinuations. Notably, MEK-inhibitors demonstrated greater efficacy in *RAS* codon 13 mutant ameloblastoma than in other tumor types, where MEK-inhibition failed^34^, suggesting a distinct pathway dependency rather than a mutation-specific effect. These therapies require careful risk-benefit evaluation, consistent with literature reporting sustained responses but frequent dose-limiting toxicities^31,32,34^.

While anecdotal reports on medication-related osteonecrosis of the jaw (MRONJ) exist for dabrafenib/ trametinib, no such associations are described for the other agents used^49–51^. Given the clinical relevance of MRONJ, all patients underwent pretherapeutic dental assessment and dental rehabilitation prior to treatment initiation; dental examinations and serial MRI imaging were performed during neoadjuvant treatment. Surgical procedures after systemic therapy were performed under antibiotic prophylaxis, sterile conditions, and with adequate wound management, and no MRONJ was observed.

Local recurrence remains a concern, with recurrence rates of 40% to 60% within six months to five years after conservative treatment^7,9^. In our cohort, no recurrences occurred following neoadjuvant therapy and enucleation over 12 months of follow-up. However, the reported recurrence dynamics of ameloblastomas clearly mandate prolonged surveillance. In contrast to patients undergoing targeted therapy, one patient who underwent primary conservative enucleation developed a locoregional recurrence.

Our findings advocate routine genomic profiling at diagnosis and prospective clinical trials evaluating neoadjuvant BRAF/MEK inhibitors in *BRAF* V600E-positive ameloblastoma, as well as targeted therapy for *FGFR2* or *HRAS* mutations. Standardized criteria for treatment protocols, therapy duration, and response assessment are needed to optimize outcomes.

The standardized precision oncology workflow in this study reinforces the clinical relevance of targeted molecularly treatment in the management of ameloblastoma. The high activity of BRAF/MEK-inhibition for *BRAF* V600E-mutated ameloblastoma supports neoadjuvant strategies to downstage tumors and convert radical surgical procedures into individualized, less morbid interventions without compromising oncologic outcome. Targeted molecular treatment beyond *BRAF* presents an innovative treatment component in refractory/recurrent ameloblastoma. Clinical trials are warranted to assess long-term outcomes of personalized therapeutic strategies.

## Methods

### Patients

Fourteen consecutively enrolled patients with histologically confirmed ameloblastoma were included in the single-center, prospective, observational precision oncology study (NCT05926284) between January 2023 and December 2024. STROBE criteria for reports of observational trials were followed (Supplemental Table S2). All were referred to the Charité Comprehensive Cancer Center for tumor genomic profiling and evaluation of targeted therapy options. Treatment allocation was non-randomized, followed the outlined precision oncology workflow and depended on availability of matched targeted therapies^25–28^. Baseline demographics and disease characteristics (age, sex, self-reported ancestry, tumor site/subsite, histologic subtype, prior ameloblastoma-directed therapy) were recorded. Diagnostic methods included clinical and dental examinations, routine imaging, and histopathological assessment by head and neck specialists. Attrition was monitored, and no patients were lost to follow-up or discontinued participation.

### Study approval

The Prospective Observational Study of Precision Medicine Approaches in Patients with Advanced Cancer (PRIME) observational study (NCT05926284) was approved by the Charité – Universitätsmedizin Berlin institutional ethics committee under EA1/021/16, with additional ethical approval obtained under EA2/019/26. All research was performed in accordance with the Declaration of Helsinki. Written informed consent for participation in the observational study was obtained from all participants. Data were pseudonymized.

### Precision oncology workflow

#### Genomic profiling

Genomic profiling of tumor tissue biopsies was routinely performed. DNA was extracted from formalin-fixed, paraffin-embedded (FFPE) sections with ≥ 20% tumor cell content using the Maxwell RSC DNA FFPE Kit (Promega GmbH, Walldorf, Germany), following manual macro-dissection of tumor areas delineated by a specialized pathologist on hematoxylin and eosin (H&E)-stained sections, by scraping the marked regions from unstained serial sections. In 12 cases, profiling was performed using the Oncomine™ Focus Assay (ThermoFisher Scientific, Life Technologies GmbH, Darmstadt, Germany), following library preparation with an input amount of 10‒100 ng DNA. This targeted next-generation sequencing (NGS) based technology detects hotspot mutations, single-nucleotide variants, and indels in 52 genes with a turnaround time of less than one week (Supplemental Table S3).

In two externally processed cases, BRAF exon 15 was assessed by melting curve analysis and Sanger sequencing, and the QIASeq Targeted DNA Panel (Qiagen, Hilden, Germany), respectively. Reported variants included oncogenic, likely pathogenic, and variants of unknown significance (VUS).

#### Molecular tumor board

Routine genomic profiling of ameloblastoma patients was clinically annotated by a specialized physician and subsequently reviewed by a molecular tumor board, resulting in recommendations for personalized treatment options. The workflow followed precision oncology guidelines^25^, with gene variant classification based on principles established by the National Center for Tumor Diseases and the Center for Personalized Medicine (NCT/ CPM)^26–28^.

#### Targeted treatment

Targeted treatment was initiated and managed following interdisciplinary board discussion. *BRAF* V600E-mutant patients received dabrafenib (150 mg twice daily) plus trametinib (2 mg daily), or dabrafenib monotherapy (150 mg twice daily). Futibatinib (20 mg once daily) was used for *FGFR2-*mutant disease, and binimetinib (45 mg twice daily) for *HRAS-*mutant disease. Treatment continued per standard dosing regimens until unacceptable toxicity or surgical resection.

### Surgical therapy

Standard virtual three-dimensional surgical planning was performed using Brainlab Elements Segmentation Cranio Facial and Object Management (Brainlab AG, Munich, Germany).

Surgical extent was based on individual tumor size and location. Advanced tumor size was treated by radical resection with 1.5–2 cm safety margins to minimize the risk of residual tumor and recurrence^9^. Segmental mandibular or extensive maxillary resections were reconstructed using free-microvascular bone grafts and patient-specific implants using computer-aided design/computer-aided manufacturing (CAD/CAM) technology during the same procedure.

A less invasive surgical approach, such as enucleation, was pursued in cases of limited disease extent, whenever feasible, to preserve healthy jawbone, teeth, nerves, and adjacent structures^52^ − particularly after targeted therapy, with low recurrence risks following conservative surgery^23^.

### Radiological assessment

Routine baseline imaging included cone beam computed tomography (CBCT), gadolinium-based magnetic resonance imaging (MRI), and panoramic radiograph. Response during molecularly matched targeted therapy was assessed by MRI following standard procedures. Radiological assessment included tumor volumetry and qualitative evaluation of bone remodeling (re-ossification) and the signal intensity of solid tumor components after gadolinium contrast agent. Tumor volume changes were assessed by comparing the MRI obtained prior to targeted therapy with either the most recent follow-up MRI or the pre-operative MRI.

RECIST 1.1 criteria, commonly applied in other solid tumors^53^, were considered unsuitable for ameloblastoma due to its distinct characteristics. Its non-metastasizing nature and typical localization in the jaw limit the applicability of strict RECIST 1.1 measurement rules – for instance, the maximum diameter of an ameloblastoma in the mandibular angle, measured in the axial plane, may not adequately reflect its size. CBCT was conducted for surgical planning following upfront targeted therapy. Postoperative follow-up MRI was performed regularly, starting six months postoperatively.

### Pathological response evaluation

Pathological response in resection specimens following targeted therapy was assessed using the College of American Pathologists (CAP) Tumor Regression Grading System, which evaluates residual viable tumor and fibrosis. Response was graded as follows: Grade 0 (complete response)—no viable tumor; Grade 1 (near complete response)—rare residual viable tumor cells; Grade 2 (partial response)—residual tumor with evident treatment effects; and Grade 3 (poor/no response)—viable tumor with minimal or no regression.

### Assessment of surgical therapy scope by resection complexity score

A resection complexity score was implemented to quantify the extent of surgical therapy based on the virtual surgical plan. It comprised five categories, each rated from 0 to 2, with a maximum total score of 10 points (Supplemental Table S1). A total score of ≤5 indicated low complexity, while >5 suggested a more extensive surgical procedure. Two oral and maxillofacial surgeons conducted an independent review, with a third surgeon resolving potential discrepancies. In patients proceeding to surgery, scores were compared between the hypothetical surgical plan prior to targeted therapy and the plan after targeted therapy to assess changes in surgical extent.

### Statistics

Data were collected in Microsoft Excel (Microsoft Corporation, Redmond, WA, USA) and analyzed using IBM SPSS Statistics Version 29 (IBM Corporation, Armonk, NY, USA). Categorical variables were reported as frequencies and percentages, while continuous variables were displayed as mean values ± standard deviation (SD) or median with interquartile range (IQR), as appropriate. Fisher’s exact test assessed mutual exclusivity of mutations and associations between ancestry, mutation status, and tumor location. Mean tumor volume reduction with 95% confidence interval was calculated using one-sample *t*-test. Postoperative follow-up was defined from surgery to last radiological imaging. Observation ended on March 1, 2025, except for four patients who received targeted therapy prior to bone-preserving enucleation, for whom follow-up was extended through March 1, 2026.

## Supplementary information

Supplementary Information

## Data Availability

The pseudonymized data supporting these findings are available within the article. Other data generated in this study are not publicly available to guard patient privacy but are available upon reasonable request from the corresponding author.
