# Supplementary material for: Targetable alterations and personalized treatment in ameloblastoma: results from a prospective observational precision oncology study
Source: NPJ Precis Oncol. 2026 Jun 12;10:217. doi: 10.1038/s41698-026-01527-6 (PMC13263350; doi:10.1038/s41698-026-01527-6)
Supplement: Supplementary file 1 — Supplementary Information [file 41698_2026_1527_MOESM1_ESM.pdf]

Supplemental Material

Supplemental Table S1.

Category	Score
**Bone resection**	
Segmental resection (maxilla: segmental or hemi-maxillectomy or opening of at least one maxillary sinus; mandible: disruption of continuity)	**2**
Non-segmental resection	**1**
No bone resection	**0**
**Soft tissue involvement**	
Extensive soft tissue resection	**2**
Fistula resection	**1**
No soft tissue resection	**0**
**Involvement of inferior alveolar nerve**	
Nerve resection	**2**
Neurolysis	**1**
No intervention on nerves	**0**
**Involvement of teeth**	
Extraction of at least two teeth	**2**
Root tip resection (apicoectomies) or extraction of one tooth	**1**
No intervention on teeth	**0**
**Surgical approach**	
Extra- and intraoral	**2**
Extraoral	**1**
Intraoral	**0**

Supplemental Table S1. Resection complexity score categories and scoring system. The resection complexity score aims to assess the extent of the surgical intervention. The table outlines the five categories, each assessed with a rating from 0 to 2, for a total score ranging from 0 to 10.

Supplemental Table S2.

	Item No	Recommendation
**Title and abstract**	**1**	(***a***) Study design is indicated in the title and abstract as "prospective observational […] study"
		**(b)** Abstract provides a balanced summary: 14 patients with ameloblastoma underwent molecular profiling; activating mutations in oncogenes were identified in all cases (BRAF, SMO, HRAS, FGFR2, PIK3CA); 11 patients received matched targeted therapies; radiological tumor regression occurred in 10/11; four underwent subsequent surgery with reduced resection extent and partial or complete pathological responses.
**Introduction**		
Background/rationale	**2**	The scientific background covers the epidemiology, histopathological subtypes, aggressive behavior, surgical morbidity, and genomic landscape of ameloblastoma, including known oncogenic drivers and their pathway dependencies. The rationale for precision oncology in this context is explained.
Objectives	**3**	The primary objective is stated as assessing radiological and pathological responses to personalized targeted therapy, particularly in the pre-surgical setting, with the aim of enabling less extensive and less morbid surgery without compromising oncologic outcome.
**Methods**		
Study design	**4**	Key elements of study design are presented early: single-center, prospective, observational study, enrolling consecutive patients with histologically confirmed ameloblastoma
Setting	**5**	Setting: Charité Comprehensive Cancer Center, Berlin, Germany. Recruitment period: January 2023 to December 2024. Data cut-off: March 1, 2025.
Participants	**6**	(***a***) Eligibility: consecutively enrolled patients with histologically confirmed ameloblastoma referred for tumor genomic profiling and evaluation of targeted therapy options. Methods of follow-up included serial MRI, clinical examinations, and postoperative imaging starting at six months postoperatively.
		(***b***) Not applicable in the sense of case-control or matched cohort design. No matching of patients to controls or exposure groups was performed.
Variables	**7**	Outcomes: radiological tumor volume reduction (MRI volumetry), pathological response (CAP Tumor Regression Grading System), and resection complexity score. Genomic alterations (mutations in BRAF, SMO, FGFR2, HRAS, PIK3CA) were the primary exposures. Diagnostic criteria for

		ameloblastoma required histological confirmation and multicystic subtype.
Data sources/ measurement	**8**	Genomic profiling was performed on FFPE tissue biopsies with ≥20% tumor cell content using the Oncomine Focus Assay (52-gene targeted NGS panel) in 12 cases; two externally profiled cases used Sanger sequencing and QIASeq Targeted DNA Panel, respectively. Radiological assessment used gadolinium-based MRI with standardized volumetry. Pathological response used the CAP grading system. Resection complexity was independently scored by two oral and maxillofacial surgeons, with a third resolving discrepancies.
Bias	**9**	Treatment allocation was non-randomized and followed a structured precision oncology workflow including molecular tumor board review. No patients were lost to follow-up. Independent surgical review for resection complexity scores was performed by two assessors with adjudication by a third.
Study size	**10**	Sample size was not formally pre-calculated; the cohort reflects consecutive enrollment over the study period (n=14) in the context of a rare tumor with an incidence of approximately 1 per million per year.
Quantitative variables	**11**	Tumor volume reduction was expressed as a percentage derived from MRI volumetry. Tumor cell content and VAF was reported as a continuous variable. Resection complexity was expressed as an ordinal score (0–10). Categorical variables were reported as frequencies and percentages; continuous variables as mean ± SD or median with IQR.
Statistical methods	**12**	(***a***) Fisher's exact test was used for mutual exclusivity of mutations and associations between ancestry, mutation status, and tumor location. Mean tumor volume reduction with 95% CI was calculated using a one-sample t-test.
		(*b*) Subgroup analysis comparing PIK3CA-comutated versus PIK3CA wild-type tumors was performed descriptively; formal testing was precluded by cohort size.
		(*c*) Missing data were present in only few cases. Missing values were not imputed.
		(*d*) No patients were lost to follow-up or discontinued participation; attrition was actively monitored.
		(*e*) No formal sensitivity analyses were performed; limitations of cohort size precluding formal statistical testing are acknowledged.

Results

Participants	**13**	(a) 14 patients were enrolled, all confirmed eligible and included in analyses. 11 received targeted therapy; 3 underwent primary surgery without systemic therapy. 4 of 11 treated patients subsequently underwent surgical resection**.**
		(b) No dropout occurred.
		(c) Flow chart (Figure 3) details the patient cohort.
Descriptive data	**14**	(a) Demographic and clinical characteristics are reported: sex, age, self-reported ancestry, histological subtype, primary and recurrence status, tumor location.
		(b) No missing data on key variables.
		(c) Postoperative follow-up time was defined from surgery to last imaging.
Outcome data	**15**	Interim radiological tumor volume reduction of 29–66% was observed in 10/11 treated patients. Among four patients undergoing surgery, pathological responses included one complete, two near-complete, and one partial remission. Resection complexity score was reduced by a median of 7 points. One local recurrence occurred in the primary surgery group.
Main results	**16**	(***a***) Tumor volume reductions are reported as individual percentages and as mean with 95% CI (51.5%; 95% CI 27.9–75.1%) for 4 neoadjuvant-treated surgical patients. No confounder adjustment was performed given the observational, descriptive nature and small cohort size.
		(***b***) Not applicable; no continuous variables were categorized.
		(***c***) Not applicable; no relative risk estimates were calculated.
Other analyses	17	Descriptive subgroup comparison of PIK3CA-comutated versus PIK3CA wild-type tumor response is reported. Mutual exclusivity of BRAF and SMO mutations was tested and found to be statistically significant (Fisher's exact test, p=0.005). Potential associations between ancestry, mutation status, and tumor location were also examined using Fisher's exact test; none reached statistical significance.

Discussion

Key results	18	Key results are summarized with reference to objectives: high frequency of targetable alterations, clinically meaningful radiological and pathological responses to matched therapies, and feasibility of neoadjuvant downsizing enabling less morbid surgery.
Limitations	19	Limitations acknowledged include the small cohort size precluding formal statistical testing of subgroup analyses, the single-center design, non-randomized treatment allocation, limited follow-up duration relative to known ameloblastoma recurrence dynamics, and heterogeneity of genomic profiling methods.
Interpretation	20	Molecularly matched therapies produce clinically meaningful responses in ameloblastoma across multiple targetable alterations, providing a strong rationale for prospective clinical trials.
Generalisability	21	Generalizability is limited by single-center setting, small cohort size, and rare tumor-specific context. Results are intended to inform trial design.

Other information

Funding	22	No funding was received for this study. No conflicts of interest were declared.

Note: An Explanation and Elaboration article discusses each checklist item and gives methodological background and published examples of transparent reporting. The STROBE checklist is best used in conjunction with this article (freely available on the Web sites of PLoS Medicine at http://www.plosmedicine.org/, Annals of Internal Medicine at http://www.annals.org/, and Epidemiology at http://www.epidem.com/). Information on the STROBE Initiative is available at www.strobe-statement.org.

Supplemental Table S2. STROBE Statement checklist for reporting observational studies.

Supplemental Table S3.

**Genes Covered by the Oncomine™ Focus Assay**<br><br>(ThermoFisher Scientific, Life Technologies GmbH, Darmstadt, Germany)
ABL1, AKT1, AKT3, ALK, AR, AXL, BRAF, CCND1, CDK4, CDK6, CTNNB1, DDR2, EGFR, ERBB2, ERBB3, ERBB4, ERG, ESR1, ETV1, ETV4, ETV5, FGFR1, FGFR2, FGFR3, GNA11, GNAQ, HRAS, MET, MTOR, MYC, MYCN, NTRK1, NTRK2, NTRK3, IDH1, IDH2, JAK1, JAK2, JAK3, KIT, KRAS, NRAS, MAP2K2, MAP2K1, PDGFRA, PIKCA, PIKC, PPARG, RAF1, RET, ROS1, SMO

Supplemental Table S3. Overview of genes covered by the Oncomine™ Focus Assay.

Supplemental Figure S1.

Patient #	Therapeutic Agent	Treatment Duration	Target Lesion Pre-Neoadjuvant	Target Lesion Post-Neoadjuvant	Tumor Volume Reduction (%)	Δ Combined Resection Complexity Score
#1	Dabrafenib + Trametinib	12 months	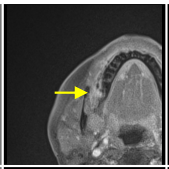	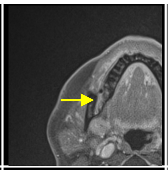	68.5%	7
#2	Dabrafenib + Trametinib	18 months	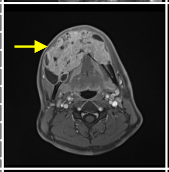	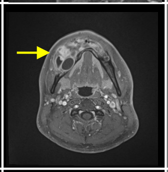	60.4%	n/a
#3	Dabrafenib + Trametinib	8 months	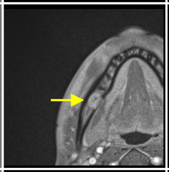	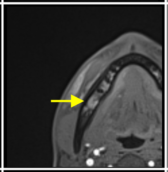	43.4%	8
#4	Dabrafenib + Trametinib	10.5 months	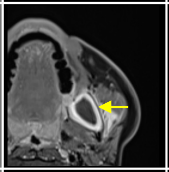	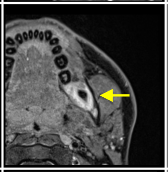	47.8%	7
#5	Dabrafenib + Trametinib	7 months	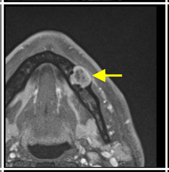	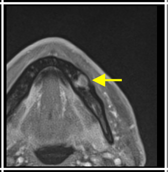	34.1%	6
#6	Dabrafenib	9 months	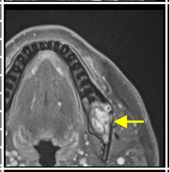	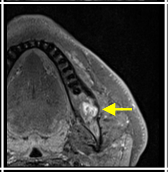	34.1%	n/a
#7	Dabrafenib + Trametinib	5 months	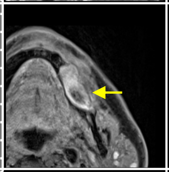	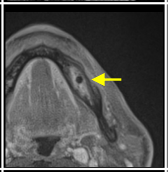	63.8%	n/a
#8	Dabrafenib + Trametinib	3 months	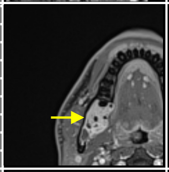	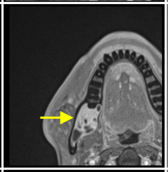	0	n/a
#9	Dabrafenib + Trametinib	3 months	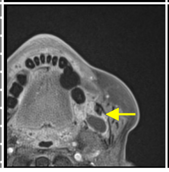	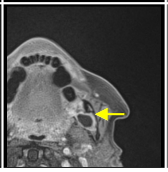	36.1%	n/a

Interim assessment – Treatment ongoing

Supplemental Figure S1. Targeted treatment details and radiological tumor visualization after BRAF-targeted therapy. The figure includes therapeutic agents, treatment duration, and MRI-based volumetry of the target lesion in the nine patients with *BRAF* V600E-mutated ameloblastoma who received or are receiving (hatched) neoadjuvant therapy. Tumor volume reduction was assessed by comparing MRI scans obtained before neoadjuvant therapy with the last MRI performed after completion. Additionally, the reduction (Δ) in the combined resection complexity after completion of targeted therapy is shown.

Supplemental Figure S2a-c.

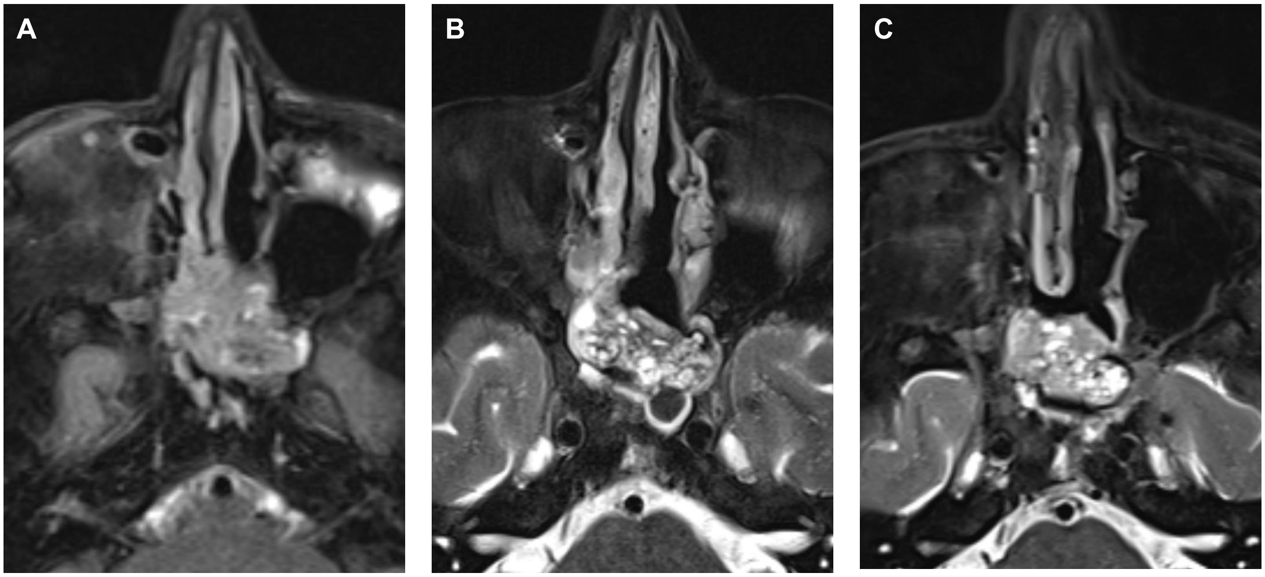

Supplemental Figure S2a-c. Radiological therapy response assessment to futibatinib in *FGFR2* C382R-mutated ameloblastoma. Fat-saturated T2-weighted MRI images show a) the tumor prior to treatment initiation, b) after treatment discontinuation, and c) at the 6-months follow-up, demonstrating a persistent reduction in tumor volume.

Supplemental Figure S3a-d.

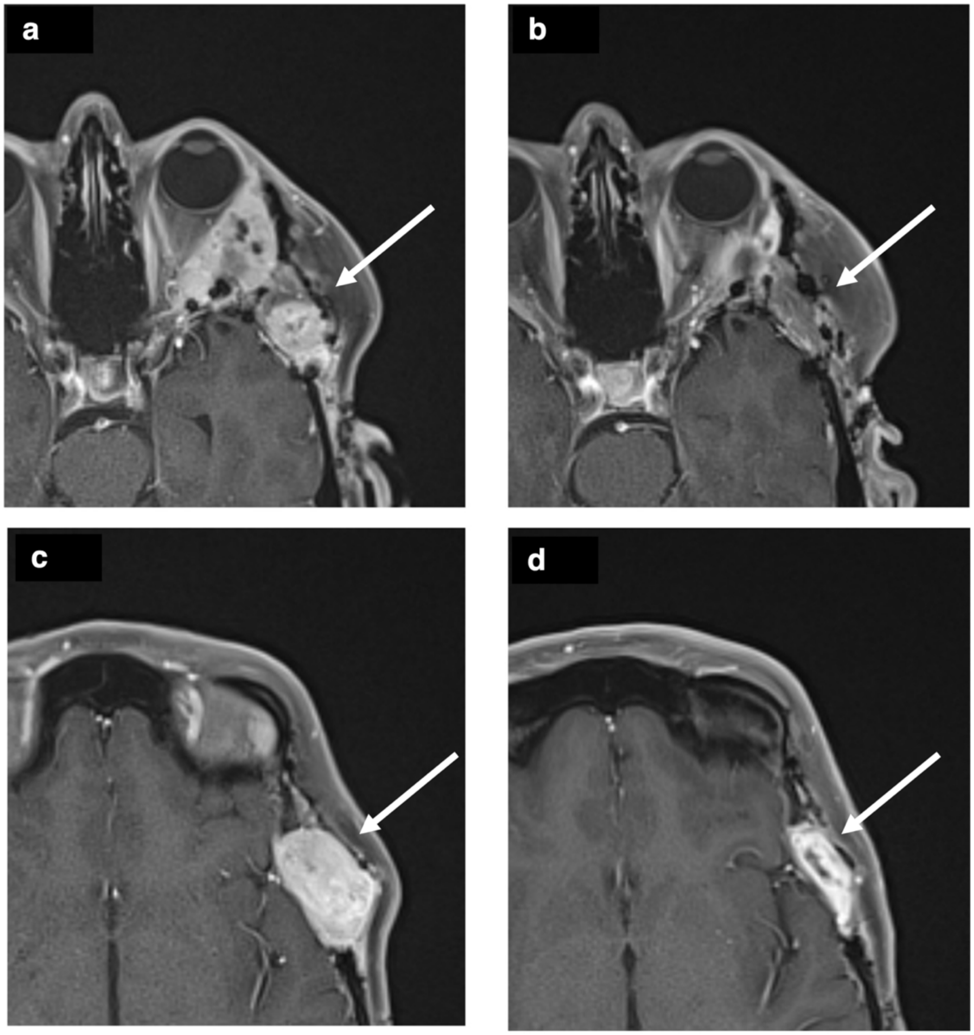

Supplemental Figure S3a-d. Radiologic response to binimetinib in an ameloblastoma harboring *HRAS* V8L (VUS) and G13R co-mutations. MRI scans show tumor (white arrow) before and after treatment for two distinct lesions: the retroorbital mass pre-treatment (a) and post-treatment (b), and the infratemporal mass pre-treatment (c) and post-treatment (d), demonstrating reduction in both lesions.
